# Venous or arterial thrombosis in COVID-19 cases in the North Carolina COVID-19 Community Research Partnership (NC-CCRP)

**DOI:** 10.1016/j.rpth.2023.100080

**Published:** 2023-02-08

**Authors:** Michael E. DeWitt, David M. Herrington, John W. Sanders, COVID-19 Community Research Partnership

**Affiliations:** 1 Department of Internal Medicine, Section on Infectious Diseases, Wake Forest University School of Medicine, Winston-Salem, North Carolina, USA; 2 Department of Internal Medicine, Section on Cardiovascular Medicine, Wake Forest University School of Medicine, Winston-Salem, North Carolina, USA

**Keywords:** cardiovascular diseases, COVID-19, prospective studies, SARS-CoV-2, thromboembolism, thrombosis

## Abstract

**Background:**

Although the incidence of venous and arterial thrombosis after a COVID-19 diagnosis and hospitalization has been well described using data available from electronic health records (EHR), little is known about their incidence after mild infections.

**Objectives:**

To characterize the cumulative incidence and risk factors for thrombosis after a COVID-19 diagnosis among those identified through the EHR and those with a self-reported case.

**Methods:**

We calculated the cumulative incidence of thromboembolism diagnoses after EHR-identified and self-reported cases in the North Carolina COVID-19 Community Partnership, a prospective, multisite, longitudinal surveillance cohort using a Kaplan-Meier approach. We performed Cox regression to estimate the hazard of a thromboembolism diagnosis after COVID-19 by comorbidities, vaccination status, and dominant SARS-CoV-2 variant.

**Results:**

Of a cohort of comprising more than 39,500 participants from 6 North Carolina sites, there were 6271 self-reported or EHR-diagnosed cases of COVID-19 reported between July 1, 2020, and April 30, 2022, of which 46 participants were diagnosed with a new-onset thromboembolism in the 365 days after their reported case. Self-reported cases had a lower estimated cumulative incidence of 0.15% (95% CI, 0.03-0.28) by day 90 and 0.64% (95% CI, 0.30-0.97) by day 365 compared with EHR-based diagnoses that had cumulative incidences of 0.73% (95% CI, 0.36-1.09) and 1.78 (95% CI, 1.14-2.46) by days 90 and 365 (log-rank test *P* value <.001). Those hospitalized and with pre-existing pulmonary and cardiovascular diseases were associated with the highest risk of a thromboembolism.

**Conclusion:**

We observed a higher cumulative incidence of thromboembolism after EHR-identified COVID-19 than self-reported cases.

## Essentials

• High rates of newly diagnosed thromboembolic diseases (TD) have been reported after COVID-19.
• We analyzed the rate of TD in self-reported and electronic health record (EHR)-identified cases.
• Self-reported COVID-19 cases have a lower incidence of TD than EHR-identified cases.
• Risk is highest in hospitalized and those with history of pulmonary and cardiovascular diseases.

## 1 Introduction

Severe COVID-19 has been associated with higher rates of thromboembolic diseases, especially among those individuals who require hospitalization [[1], [2], [3]]. However, most SARS-CoV-2 infections, the causative agent of COVID-19, do not progress to hospitalization, especially among the vaccinated [[4], [5], [6]]. Recent work by Burn et al. [7] found that the estimated risk of venous and arterial embolism was nearly 1% for all COVID-19 cases, not limited to hospitalizations, in a retrospective study of more than 900,000 electronic health records (EHR) from 5 European countries after September 1, 2020. Similarly, a large study by Knight and colleagues using EHR records of 48 million adults in England and Wales found among those diagnosed with COVID-19 were 34% and 80% more likely to be diagnosed with arterial and venous thromboses, respectively, by weeks 27 to 49 when compared with those without a COVID-19 diagnosis. Because these findings relied on EHR records, patients with pauci-symptomatic and mild infections who did not seek treatment were likely underrepresented.

In this study, we describe the cumulative incidence of thromboembolic diseases after COVID-19 infections among those who self-reported a COVID-19 infection vs those who were identified through the EHR using the North Carolina COVID-19 Community Research Partnership (NC-CCRP), a well-defined, prospective cohort of more than 39,500 adult participants [8]. The NC-CCRP provides insight into the incidence and associated sequelae of COVID-19 cases that may not be captured in the EHR. We explored the association of comorbidities, dominant variant, and vaccination status with the risk of a thrombotic disease after infection. An additional secondary analysis included a self-controlled case series design among those in the cohort with thromboembolism diagnosis since January 1, 2020, to estimate the incidence ratio before and after COVID-19 diagnoses with each participant serving as their own control.

## 2 Methods

### 2.1 Study design

The NC-CCRP is prospective cohort of more than 39,500 adult participants who completed daily health and symptom logs from April 1, 2020, through April 30, 2022 [9]. Adults 18 years and older were recruited via direct email outreach from the patient populations served by healthcare systems at 6 North Carolina sites. Participants provided informed consent electronically. Participants received a daily electronic survey via text or email to answer questions about COVID-19 exposures, symptoms, test results, receipt of vaccination, and risk behaviors. Demographic information was obtained at baseline, and medical history was extracted from the EHR. The NC-CCRP is an observational, longitudinal, multisite surveillance study approved by the institutional review board for Wake Forest University Health Sciences.

Self-reported positive COVID-19 diagnosis was defined as a study participant answering that they had a positive “COVID-19 infection (nasal swab, saliva, or spit)” as recorded by the daily syndromic survey. An EHR-confirmed COVID-19 diagnosis was defined as a recorded EHR-confirmed positive laboratory result or a diagnosis of COVID-19 (defined as presence of *International Statistical Classification of Diseases and Related Health Problems Revision 10* [ICD-10]; Supplementary Table 1). In the case of multiple records, the first date was used (eg, first of self-reported positive or EHR record). Vaccination status at date of reported COVID-19 was recorded. SARS-CoV-2 variant waves were determined by examining the periods in which a given variant was the predominant sequence reported by the North Carolina Department of Health and Human Services. We defined pre-Delta as an infection before June 26, 2021. Delta was the predominant variant from June 26 to December 25, 2021, after which Omicron became dominant [10]. We identified diagnoses of arterial and venous thromboembolisms using ICD-10 codes available within the EHR, consistent with previous studies [7,11,12]. A full list of the codes used to identify venous and arterial thromboembolic events are included in the appendix (Supplementary Table 1).

Comorbidities were defined using Healthcare Cost and Utilization Project codes using Centers for Disease Control and Prevention definitions [13]. Comorbidities included presence of an autoimmune disease, cancer (excluding some skin cancers, myelodysplastic syndrome, and benign neoplasms), immune compromising conditions (includes HIV infection), liver disease, mental health conditions, neurologic conditions, obesity, and pulmonary disease.

The analysis cohort consisted of those NC-CCRP participants who had a self-reported or EHR-identified case of COVID-19 between July 1, 2020, and April 30, 2022. Those with a history of thromboembolism (defined as a diagnosis of a thromboembolism up to 365 days before the date of COVID-19) were excluded. A secondary analysis cohort was generated for a self-controlled case series design from participants who reported a COVID-19 diagnosis and a new diagnosis of thromboembolism, where new represented no thromboembolism diagnosis in the previous year, after January 1, 2020. The exposure period was defined as the date of COVID-19 report up to 365 days. Participants who could not be associated with their EHR were excluded from the analysis.

### 2.2 Outcome

A participant was considered to have a new thromboembolic diagnosis if the participant had an EHR record of a thromboembolic diagnosis between the first reported date of COVID-19 up to 365 days after first reported date. The date of the first thromboembolism diagnosis was considered the time of the event. Participants were censored in the event of death, at the end of 365 days of follow-up or the end of the study period, whichever occurred sooner. The primary outcome of interest was the cumulative incidence of new thromboembolic diagnosis by the COVID-19 diagnosis method. The secondary outcomes were the associated hazard of thromboembolic diagnosis by comorbidity, variant period, vaccination status at the time of infection, hospitalization with COVID-19, and the incidence ratio of thromboembolism after COVID-19 in the self-controlled case series.

### 2.3 Statistical methods

We used a Kaplan-Meier approach to estimate the cumulative incidence of new thromboembolism diagnoses overall and by diagnosis source. A log-rank test was used to detect whether a statistically significant difference was observed between the sources of COVID-19 diagnosis. We performed adjusted and unadjusted Cox regressions to estimate the association of comorbidities, vaccination status at time of the infection, and variant period on the risk of new thromboembolism. Age, sex, and hospitalization status were included as covariates in the adjusted model as potential sources of confounding. Schoenfeld residuals were plotted to visually confirm that proportional hazards assumption was not violated. Incidence rate ratios (IRR) were calculated using conditional Poisson regression models to compare the intraperson incidence rates of thromboembolism after COVID-19 in days 1 to 365 after diagnosis with the baseline (pre-COVID-19) period. We considered *P* values <.05 statistically significant.

All analysis performed using R version 4.1.3 (2022-03-10).

## 3 Results

Among the 6271 self-reported or EHR-diagnosed cases of COVID-19 reported between July 1, 2020, and April 30, 2022, 46 study participants were diagnosed with a new-onset thromboembolism in the 365 days after their reported case date (Table 1). Those that were diagnosed with a thromboembolism tended to be older than those who were not with a median age of 58 years (IQR, 48-67) vs 48 years (IQR, 38-59). The estimated overall cumulative incidence was 0.35% (95% CI, 0.20-0.50) by day 90 and 1.09% (95% CI, 0.75-1.41) by day 365 after reported COVID-19 case (Supplementary Fig. 1).

**Table 1:** Key demographics and characteristics of the study cohort

Characteristic	Entire study cohort			New-onset thromboembolism		
	No thromboembolism *N* = 6225	New thromboembolism *N* = 46	*P* value^a^	EHR identified *N* = 30	Self-reported *N* = 16	*P* value^a^
Age (y), median (IQR)	48 (38-59)	58 (48-67)	<.001	54 (42-65)	64 (60-72)	.021
Race, *n* (%)			.087			.08
Black or African American	287 (4.6)	5 (11)		5 (17)	0 (0)	
White	5559 (89)	40 (87)		25 (83)	15 (94)	
Other^b^	379 (6.1)	1 (2.2)		0 (0)	1 (6.2)	
Sex, *n* (%)			.92			.3
Women	4507 (72)	33 (72)		23 (77)	10 (62)	
Men	1718 (28)	13 (28)		7 (23)	6 (38)	
COVID-19 report source						
EHR	2047 (33)	30 (65)		30 (100)	-	
Self-reported	4178 (67)	16 (35)		-	16 (100)	
Status at infection, *n* (%)			.042			.12
Unvaccinated	2911 (47)	30 (65)		22 (73)	8 (50)	
Vaccinated^b^	1595 (26)	7 (15)		2 (6.7)	5 (31)	
Boosted^c^	1719 (28)	9 (20)		6 (20)	3 (19)	
Autoimmune condition, *n* (%)	1256 (20)	22 (48)	<.001	13 (43)	9 (56)	.4
Cardiovascular disease, *n* (%)	1965 (32)	36 (78)	<.001	22 (73)	14 (88)	.5
Diabetes, *n* (%)	573 (9.2)	15 (33)	<.001	10 (33)	5 (31)	.9
Obesity, *n* (%)	1282 (21)	17 (37)	.006	9 (30)	8 (50)	.2
Pulmonary disease, *n* (%)	809 (13)	26 (57)	<.001	17 (57)	9 (56)	>.9
Renal disease, *n* (%)	127 (2.0)	6 (13)	<.001	4 (13)	2 (12)	>.9
Immunocompromised, *n* (%)	116 (1.9)	3 (6.5)	.056	2 (6.7)	1 (6.2)	>.9
Cancer, *n* (%)	419 (6.7)	4 (8.7)	.55	2 (6.7)	2 (12)	.6
Liver disease, *n* (%)	184 (3.0)	2 (4.3)	.40	2 (6.7)	0 (0)	.5
Mental disorder, *n* (%)	931 (15)	8 (17)	.64	6 (20)	2 (12)	.7
Neurologic disorder, *n* (%)	1262 (20)	25 (54)	<.001	18 (60)	7 (44)	.3
Substance abuse disorder, *n* (%)	349 (5.6)	8 (17)	.004	7 (23)	1 (6.2)	.2
Predominant variant during infection, *n* (%)			.012			.1
Pre-Delta	2595 (42)	28 (61)		20 (67)	8 (50)	
Delta	1133 (18)	9 (20)		3 (10)	6 (38)	
Omicron	2497 (40)	9 (20)		7 (23)	2 (12)	

EHR, electronic health records.

a Pearson’s chi-squared test; Kruskal-Wallis rank sum test; Fisher exact test.

b A participant was considered vaccinated during their case if the reporting date was after their fully vaccinated date (defined as 14 days after the second mRNA vaccine or 14 days after one dose of a viral vectored vaccine).

c A participant was considered boosted after receiving their appropriate booster dose (date of receipt of second dose at least 60 days following a viral vectored vaccine or date of receipt of third dose of mRNA vaccine at least 60 days following receipt of second dose).

**Figure 1:**
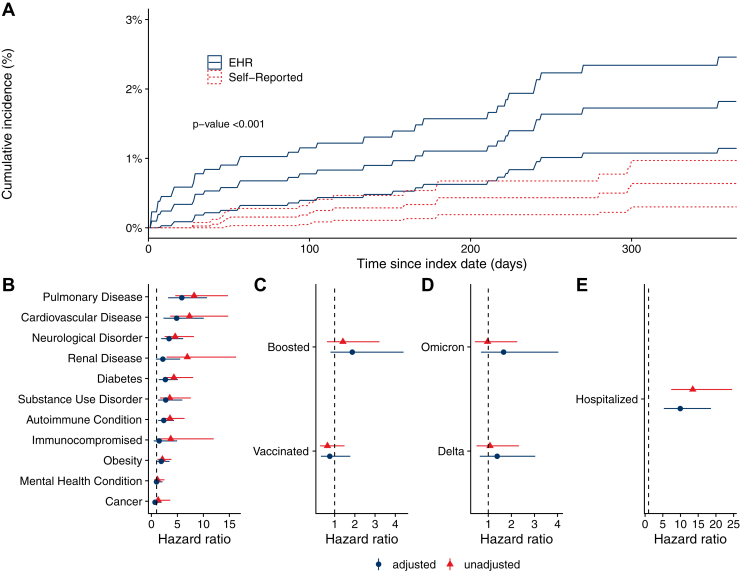
The estimate cumulative incidence of thromboembolism diagnosis by source of reported COVID-19 (A). Estimated hazard ratios for thromboembolism diagnosis by comorbidity condition (B). Vaccination status, reference is unvaccinated (C). Predominant SARS-CoV-2 variant at the time of infection, reference is pre-Delta (D). Adjusted hazards account for the contribution of age, sex, and hospitalization. Estimated hazard ratios for thromboembolism diagnosis by hospitalization status (E), reference is nonhospitalized; adjusted hazards account for the contribution of age and sex

When considering the source of COVID-19 diagnosis, self-reported cases had a lower estimated cumulative incidence of 0.15% (95% CI, 0.03-0.28) by day 90 and 0.64% (95% CI, 0.30-0.97) by day 365 compared with EHR-based diagnoses which had cumulative incidences of 0.73% (95% CI, 0.36-1.09) and 1.78 (95% CI, 1.14-2.46) by days 90 and 365, respectively (Figure 1, panel A). These cumulative incidences translate to 1996 events per 100 000 person years for those cases identified through the EHR and 676 events per 100,000 person years among the self-reported cases for an overall rate of 1188 events per 100,000 person years (Supplementary Table 2). The log-rank test indicates that there was a statistically significant difference detected between those who self-reported COVID-19 vs those who were identified through the EHR (*P* <.001, Figure 1, panel A).

Histories of pulmonary and cardiovascular diseases were associated with the highest risk of a thromboembolism diagnosis with adjust hazard ratios of 5.86 (95% CI, 3.20-10.71) and 4.85 (95% CI, 2.33-10.08), respectively (Figure 1, panel B). An immunocompromising condition, mental health conditions, and cancer were not associated with an increased risk of diagnosis of a thromboembolism after COVID-19. We failed to detect a statistically significant difference among the different vaccination statuses or the different variants (Figure 1, panels C and D).

In our self-controlled case series design, 105 participants were diagnosed with thromboembolism and met the inclusion criteria for analysis. Compared with the baseline period, exposure to COVID-19 was associated with an IRR of 1.54 (95% CI, 1.03-2.32) for all cases. The IRR of thromboembolism was 1.92 times higher (95% CI, 1.12-3.31) than baseline when considering only EHR-identified COVID-19, whereas we failed to detect a statistically significant increase in risk associated with self-reported COVID-19 with an IRR of 1.15 (95% CI, 0.61-2.19; Supplementary Fig. 2).

## 4 Discussion and Conclusion

In this study, we find that the cumulative incidence of venous and arterial thromboembolisms is lower among those who self-reported a COVID-19 diagnosis than those who had a COVID-19 diagnosis identified through the EHR. These findings supplement those of Burn and colleagues as well as those by Knight and colleagues by including those COVID-19 cases that may have been underascertained when using EHR data. These findings continue to underline the association of thromboembolic disease after COVID-19 and support a growing body of literature that suggest higher incidence of thrombotic disease postinfection [1,7,12,[14], [15], [16], [17]].

Those who were diagnosed with a thromboembolism tended to be older than those who were not in agreement with studies suggesting the association of increased age with baseline risk of thromboembolism [18]. Underlying comorbidities, especially those with underlying cardiovascular and pulmonary diseases and diabetes, have been associated with progression to hospitalization in COVID-19 cases [[19], [20], [21], [22]] The associations of these comorbidities with a subsequent thromboembolism diagnosis remained even after adjusting for age and sex. Hospitalization for COVID-19 was associated with the highest risk of a subsequent thromboembolism. Those with EHR-identified cases may have been more likely to have more severe disease and thus were at higher risk for adverse outcomes. Furthermore, analysis that relies on EHR-identified cases may generate biased estimates of the rate of adverse outcomes because of this association. Our results regarding self-reported COVID-19 cases align well with several retrospective studies that have shown lower rates of thromboembolism among those with milder COVID-19 compared with those who require hospitalization [[23], [24], [25]]. Furthermore, our results support the role of thromboprophylaxis for those hospitalized with COVID-19 [26].

Although vaccination has been shown to reduce the risk of progression to severe disease and hospitalization with reduced incidence of sequela [[27], [28], [29]], this study failed to detect a difference in the risk of thrombotic diseases after infection because of small sample sizes. Similarly, we could not explore the interaction of different comorbidities and their effect on subsequent thromboembolism diagnoses owing to small sample sizes.

This study has several key limitations. As a convenience, prospective cohort, the study population may not generalize to a larger population. Treatment sought by participants outside of the EHR (eg, free clinics) was not recorded and could underrepresent the incidence of thromboembolism after infection. This study relies on the completeness and accuracy of diagnosis codes. Patients with a history of thromboembolisms, have known comorbidities (eg, cardiovascular disease), or are in a more acute disease state may be more likely to be assessed for thromboembolism after COVID-19 leading to higher ascertainment rates in these groups. Heterogeneous access to preventive care during mild infections may have an impact on the incidence of thromboembolism. Small sample sizes in the cohort limited the statistical power to detect differences in hazards among the vaccinated and boosted or the ability to explore interactions among the comorbidities and vaccination status. In addition to the small sample size, our self-controlled case series does not account for changing behaviors within an individual during the pandemic (eg, increased propensity for air travel after COVID-19), which may impact the likelihood of a thromboembolism.

The high global burden of infection continues to underline the importance of recognition and treatment strategies for COVID-19 sequela. Self-reported infections were associated with a lower cumulative incidence of thrombotic disease than EHR-identified diagnoses for COVID-19, and a self-controlled case series approach failed to detect a statistically significant difference in the incidence ratio of thrombotic diseases among those self-reporting COVID-19 compared with the pre-COVID-19 control period.
