# Supplementary material for: Venous or arterial thrombosis in COVID-19 cases in the North Carolina COVID-19 Community Research Partnership (NC-CCRP)
Source: Res Pract Thromb Haemost. 2023 Feb 8;7(2):100080. doi: 10.1016/j.rpth.2023.100080 (PMC9904855; doi:10.1016/j.rpth.2023.100080)
Supplement: Appendix [file mmc1.docx]

**Appendix: Venous or Arterial Thrombosis in COVID-19 Cases in the North Carolina COVID-19 Community Research Partnership (NC-CCRP)**

**Table S1:** ICD-10 codes utilized to determine thromboembolic events in the study population

| **Outcome** | **ICD-10** |
| --- | --- |
| EHR COVID-19 Diagnosis | B97.29 between January 18, 2020, and April 1, 2020; U07.1 after April 1, 2020; B97.21 after January 18, 2020; or J12.82 after January 18, 2020 |
| Arterial thromboembolic events | I21, I22, I23, I24, G95.1, I63.1, I63.8, I63.3, I64, I63.4, I63.2, I63.9, I63.5, I63.0, H34, I74 |
| Venous thromboembolic events | G08, I26.0, I26.9, I63.6, I67.6, I80, I81, I82.0, I82.2, I82.3, I82.8, I82.9, O87.1, O87.9, O88.2, O22.3, O87.3, O22.5 |
| Note: ICD-10 codes were mixtures of primary and non-primary positions | |

**Table S2.** Thromboembolism Diagnoses per 100 000 person years

| **Strata** | **Events (N)** | **Events per 100 000 person years** |
| --- | --- | --- |
| EHR Diagnosed | 30 | 1996 |
| Self-Reported | 16 | 676 |
| Overall | 46 | 1188 |

**Table S3.** Incidence Ratios of Thromboembolism Diagnosis After COVID-19 Diagnosis Overall and by Reporting Source using Self-Controlled Case Series Design

| **Period** | **All** | | **EHR Diagnosed** | | **Self-Reported** | |
| --- | --- | --- | --- | --- | --- | --- |
|  | No. of Cases  (Person-Years) | IR (95% CI)^1^ | No. of Cases  (Person-Years) | IR (95% CI) ^1^ | No. of Cases  (Person-Years) | IR (95% CI) ^1^ |
| 1-365 Days | 43 (77.2) | 1.54  (1.03-2.32) | 28 (45.4) | 1.92  (1.12-3.31) | 15 (31.8) | 1.15  (0.61-2.19) |
| Baseline | 64 (77.2) | 1.00 | 30 (88.0) | 1.00 | 34 (82.2) | 1.00 |
| IR denotes incidence ratio utilizing the baseline period (pre-COVID-19 infection) as reference, and CI confidence interval. Note that these data represent 105 unique individuals. Two individuals were diagnosed with a thromboembolism greater than 365 days apart and are those present twice in the data. | | | | | | |

**Figure S1.** Overall Cumulative Incidence

**
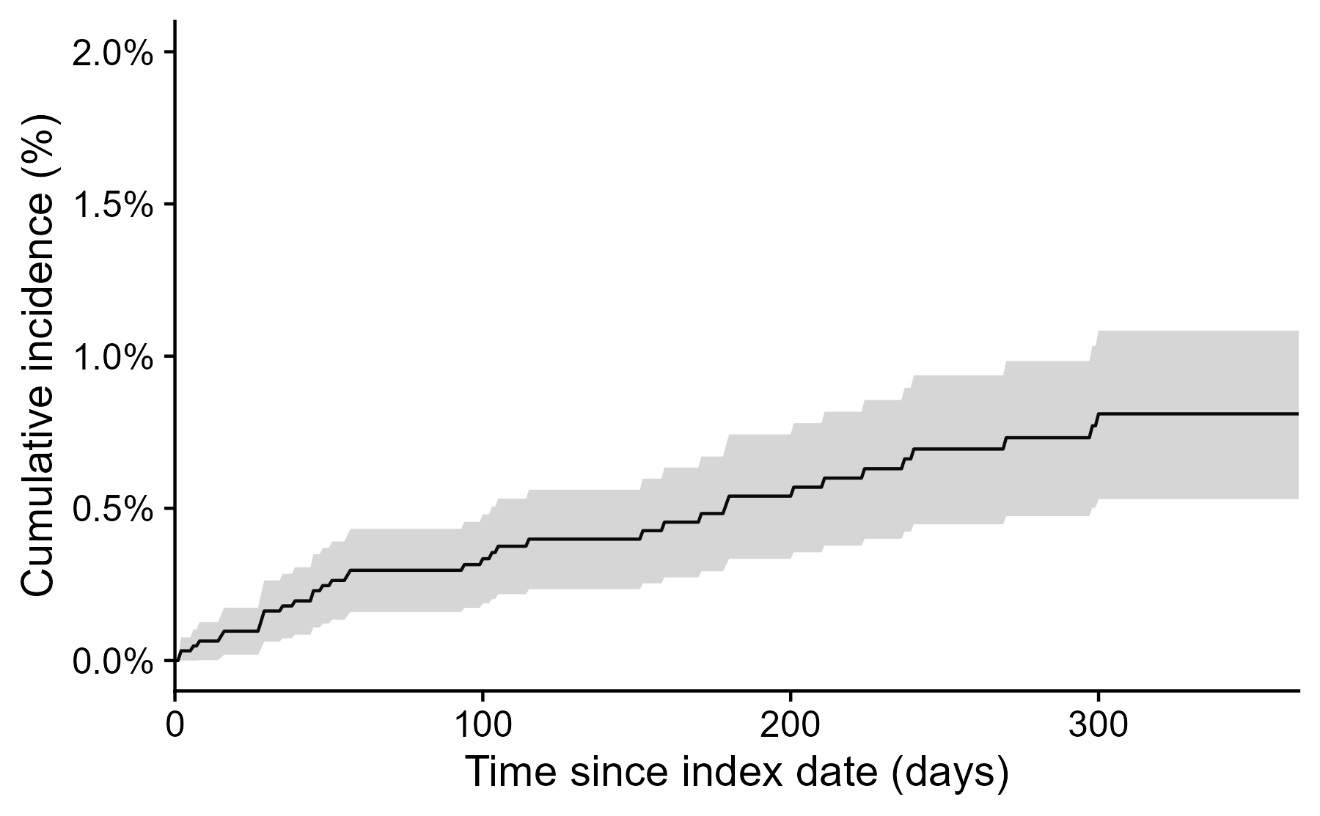
**

**Figure S2.** Incidence ratio of thromboembolism diagnosis after COVID-19 diagnosis up to 365 days after reported case compared to the pre-COVID-19 period amongst those participants with a new thromboembolism in the two years prior to reported infection. Dashed line represents baseline (pre-COVID-19 diagnosis).

**
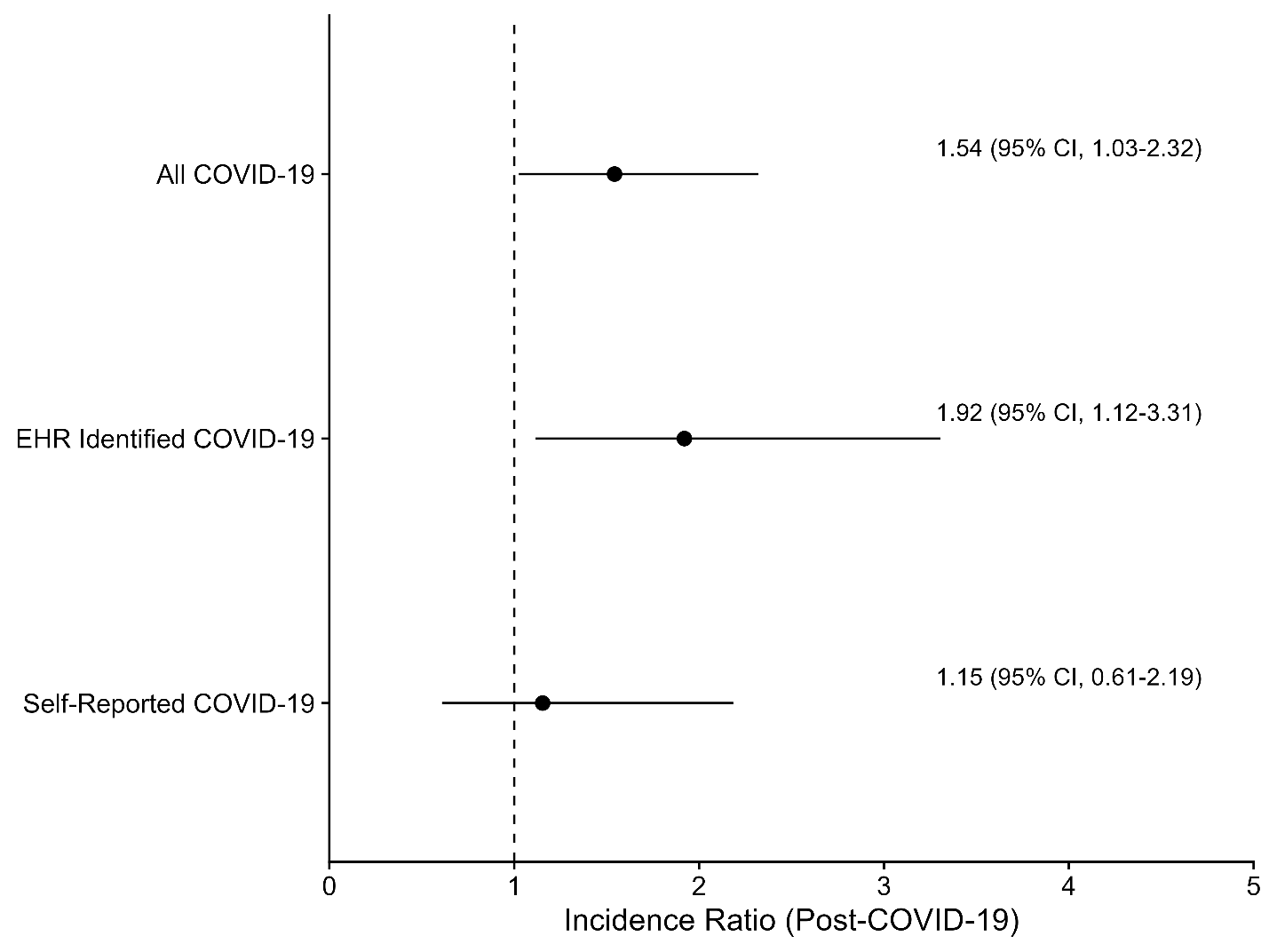
**

**Funding**

CARES Act of the U.S. Department of Health and Human Services through the State of North Carolina [Contract # NC DHHS GTS #49927]. Funders had no in study design; collection, analysis, and interpretation of data; in writing the report; and in the decision to submit this correspondence for publication.

**COVID-19 Community Research Partnership***The COVID-19 Research Group (*Site Principal Investigator)*

*Wake Forest School of Medicine*: Thomas F Wierzba PhD, MPH, MS*, John Walton Sanders, MD, MPH, David Herrington, MD, MHS, Mark A. Espeland, PhD, MA, John Williamson, PharmD, Morgana Mongraw-Chaffin, PhD, MPH, Alain Bertoni, MD, MPH, Martha A. Alexander-Miller, PhD, Paola Castri, MD, PhD, Allison Mathews, PhD, MA, Iqra Munawar, MS, Austin Lyles Seals, MS, Brian Ostasiewski, Christine Ann Pittman Ballard, MPH, Metin Gurcan, PhD, MS, Alexander Ivanov, MD, Giselle Melendez Zapata, MD, Marlena Westcott, PhD, Karen Blinson, Laura Blinson, Mark Mistysyn, Donna Davis, Lynda Doomy, Perrin Henderson, MS, Alicia Jessup, Kimberly Lane, Beverly Levine, PhD, Jessica McCanless, MS, Sharon McDaniel, Kathryn Melius, MS, Christine O’Neill, Angelina Pack, RN, Ritu Rathee, RN, Scott Rushing, Jennifer Sheets, Sandra Soots, RN, Michele Wall, Samantha Wheeler, John White, Lisa Wilkerson, Rebekah Wilson, Kenneth Wilson, Deb Burcombe, Georgia Saylor, Megan Lunn, Karina Ordonez, Ashley O’Steen, MS, Leigh Wagner.

*Atrium Health*: Michael S. Runyon MD, MPH*, Lewis H. McCurdy MD*, Michael A. Gibbs, MD, Yhenneko J. Taylor, PhD, Lydia Calamari, MD, Hazel Tapp, PhD, Amina Ahmed, MD, Michael Brennan, DDS, Lindsay Munn, PhD RN, Keerti L. Dantuluri, MD, Timothy Hetherington, MS, Lauren C. Lu, Connell Dunn, Melanie Hogg, MS, CCRA, Andrea Price, Marina Leonidas, Melinda Manning, Whitney Rossman, MS, Frank X. Gohs, MS, Anna Harris, MPH, Jennifer S. Priem, PhD, MA, Pilar Tochiki, Nicole Wellinsky, Crystal Silva, Tom Ludden PhD, Jackeline Hernandez, MD, Kennisha Spencer, Laura McAlister.

*MedStar Health Research Institute*: William Weintraub MD*, Kristen Miller, DrPH, CPPS*, Chris Washington, Allison Moses, Sarahfaye Dolman, Julissa Zelaya-Portillo, John Erkus, Joseph Blumenthal, Ronald E. Romero Barrientos, Sonita Bennett, Shrenik Shah, Shrey Mathur, Christian Boxley, Paul Kolm, PhD, Ella Franklin, Naheed Ahmed, Moira Larsen.

*Tulane*: Richard Oberhelman MD*, Joseph Keating PhD*, Patricia Kissinger, PhD, John Schieffelin, MD, Joshua Yukich, PhD, Andrew Beron, MPH, Johanna Teigen, MPH.

*University of Maryland School of Medicine*: Karen Kotloff MD*, Wilbur H. Chen MD, MS*, DeAnna Friedman-Klabanoff, MD, Andrea A. Berry, MD, Helen Powell, PhD, Lynnee Roane, MS, RN, Reva Datar, MPH, Colleen Reilly.

*University of Mississippi*: Adolfo Correa MD, PhD*, Bhagyashri Navalkele, MD, Yuan-I Min, PhD, Alexandra Castillo, MPH, Lori Ward, PhD, MS, Robert P. Santos, MD, MSCS, Pramod Anugu, Yan Gao, MPH, Jason Green, Ramona Sandlin, RHIA, Donald Moore, MS, Lemichal Drake, Dorothy Horton, RN, Kendra L. Johnson, MPH, Michael Stover.

*Wake Med Health and Hospitals*: William H. Lagarde MD*, LaMonica Daniel, BSCR.

*New Hanover*: Patrick D. Maguire MD*, Charin L. Hanlon, MD, Lynette McFayden, MSN, CCRP, Isaura Rigo, MD, Kelli Hines, BS, Lindsay Smith, BA, Monique Harris, CCRP, Belinda Lissor, AAS, CCRP, Vivian Cook, MA, MPH, Maddy Eversole, BS, Terry Herrin, BS, Dennis Murphy, RN, Lauren Kinney, BS, Polly Diehl, MS, RHIA, Nicholas Abromitis, BS, Tina St. Pierre, BS, Bill Heckman, Denise Evans, Julian March, BA, Ben Whitlock, CPA, MSA, Wendy Moore, BS, AAS, Sarah Arthur, MSW, LCSW, Joseph Conway.

*Vidant Health*: Thomas R. Gallaher MD*, Mathew Johanson, MHA, CHFP, Sawyer Brown, MHA, Tina Dixon, MPA, Martha Reavis, Shakira Henderson, PhD, DNP, MS, MPH, Michael Zimmer, PhD, Danielle Oliver, Kasheta Jackson, DNP, RN, Monica Menon, MHA, Brandon Bishop, MHA, Rachel Roeth, MHA.

*Campbell University School of Osteopathic Medicine*: Robin King-Thiele DO*, Terri S. Hamrick PhD*, Abdalla Ihmeidan, MHA, Amy Hinkelman, PhD, Chika Okafor, MD (Cape Fear Valley Medical Center), Regina B. Bray Brown, MD, Amber Brewster, MD, Danius Bouyi, DO, Katrina Lamont, MD, Kazumi Yoshinaga, DO, (Harnett Health System), Poornima Vinod, MD, A. Suman Peela, MD, Giera Denbel, MD, Jason Lo, MD, Mariam Mayet-Khan, DO, Akash Mittal, DO, Reena Motwani, MD, Mohamed Raafat, MD (Southeastern Health System), Evan Schultz, DO, Aderson Joseph, MD, Aalok Parkeh, DO, Dhara Patel, MD, Babar Afridi, DO (Cumberland County Hospital System, Cape Fear Valley).

*George Washington University Data Coordinating Center*: Diane Uschner PhD*, Sharon L. Edelstein, ScM, Michele Santacatterina, PhD, Greg Strylewicz, PhD, Brian Burke, MS, Mihili Gunaratne, MPH, Meghan Turney, MA, Shirley Qin Zhou, MS, Ashley H Tjaden, MPH, Lida Fette, MS, Asare Buahin, Matthew Bott, Sophia Graziani, Ashvi Soni, MS, Guoqing Diao, PhD, Jone Renteria, MS.

*George Washington University Mores Lab*: Christopher Mores, PhD, Abigail Porzucek, MS.

*Oracle Corporation*: Rebecca Laborde, Pranav Acharya.

*Sneez LLC*: Lucy Guill, MBA, Danielle Lamphier, MBA, Anna Schaefer, MSM, William M. Satterwhite, JD, MD.

*Vysnova Partners*: Anne McKeague, PhD, Johnathan Ward, MS, Diana P. Naranjo, MA, Nana Darko, MPH, Kimberly Castellon, BS, Ryan Brink, MSCM, Haris Shehzad, MS, Derek Kuprianov, Douglas McGlasson, MBA, Devin Hayes, BS, Sierra Edwards, MS, Stephane Daphnis, MBA, Britnee Todd, BS.

*Javara Inc*: Atira Goodwin.

*External Advisory Council*: Ruth Berkelman, MD, Emory, Kimberly Hanson, MD, U of Utah, Scott Zeger, PhD, Johns Hopkins, Cavan Reilly, PhD, U. of Minnesota, Kathy Edwards, MD, Vanderbilt, Helene Gayle, MD MPH, Chicago Community Trust, Stephen Redd.

**Acknowledgements**

The COVID-19 Community Research Partnership gratefully acknowledges the commitment and dedication of the study participants. Programmatic, laboratory and technical support was provided by Vysnova Partners, Inc., Oracle, Scanwell Health, and Neoteryx. This publication is supported by the CARES Act, of the U.S. Department of Health and Human Services (HHS) [Contract # NC DHHS GTS #49927]. The Partnership is listed in clinicaltrials.gov (NCT04342884). The contents are those of the author(s) and do not necessarily represent the official views of, nor an endorsement, by HHS, or the U.S. Government.
